# Tackling Age of Information in Access Policies for Sensing Ecosystems †

**DOI:** 10.3390/s23073456

**Published:** 2023-03-25

**Authors:** Alberto Zancanaro, Giulia Cisotto, Leonardo Badia

**Affiliations:** 1 Department Information Engineering, University of Padova, Via Gradenigo, 6/b, 35121 Padova, Italy; giulia.cisotto@unimib.it (G.C.); leonardo.badia@unipd.it (L.B.); 2 Department Informatics, Systems, and Communication, University of Milan-Bicocca, Viale Sarca 336, 20126 Milan, Italy

**Keywords:** age of information, Internet of Things, data acquisition, networks, machine learning

## Abstract

Recent technological advancements such as the Internet of Things (IoT) and machine learning (ML) can lead to a massive data generation in smart environments, where multiple sensors can be used to monitor a large number of processes through a wireless sensor network (WSN). This poses new challenges for the extraction and interpretation of meaningful data. In this spirit, age of information (AoI) represents an important metric to quantify the freshness of the data monitored to check for anomalies and operate adaptive control. However, AoI typically assumes a binary representation of the information, which is actually multi-structured. Thus, deep semantic aspects may be lost. In addition, the ambient correlation of multiple sensors may not be taken into account and exploited. To analyze these issues, we study how correlation affects AoI for multiple sensors under two scenarios of (i) concurrent and (ii) time-division multiple access. We show that correlation among sensors improves AoI if concurrent transmissions are allowed, whereas the benefits are much more limited in a time-division scenario. Furthermore, we discuss how ML can be applied to extract relevant information from data and show how it can further optimize the transmission policy with savings of resources. Specifically, we demonstrate, through simulations, that ML techniques can be used to reduce the number of transmissions and that classification errors have no influence on the AoI of the system.

## 1. Introduction

The last decade has seen unprecedented development in smart environments due to the technological advancements in the IoT, sensors, and artificial intelligence. There is a wide gamma of applications for these innovations in smart living environments, from smart houses to assisted living, especially for elderly people [1,2]. In addition, IoT techniques contribute to achieving better sustainable energy consumption [3], and the introduction of these solutions for sensing, data analysis, and active system control enables the creation of smart cyber-physical ecosystems, where machine and people are interconnected [4]. Such new technologies also lead to a tremendous increase in the amount of data produced and consequently hinder their management [5]. Specifically, one of the most used technologies are the WSN. WSN are widely exploited to monitor smart living environments (e.g., houses, airports, industries, hospitals where they are used for constant monitoring, continuously collect data and transmit information of the current status of the environment.

In this scenario, AoI represents an important metric to quantify the freshness of data coming from real-time monitoring of status updates or control [6,7]. This implies that it is possible to improve the sensor’s battery consumption and the use of the network communication bandwidth according to the freshness of data and the degree of innovation they bring to the historical description of the cyber–physical ecosystem.

Over the years, different approaches have been proposed to optimize various network features with an eye to AoI as a key performance indicator. For example, ref. [8] optimizes transmission and sampling cost in a wireless network under AoI constraints through Lyapunov optimization theory. In [9], game theory is used to minimize the AoI from two different competing sources. Another area where AoI is becoming increasingly important is energy optimization, such as in [10,11,12], where the problem of assessing the impact of energy harvesting on AoI is analyzed.

A factor that limits the use of the AoI is the simplicity of the metric, which encodes only the freshness of the information, but not the semantic value it can have within complex scenarios. From the point of view of the AoI an update due to an anomaly or a routine update have the same importance, which is undesirable whenever the scenario is supposed to provide some application in a smart living context. Correlation among multiple neighboring sources [13,14,15,16] is another important factor to consider when taking AoI into account since an update could also deliver extra information related to other data sources. This occurs especially in the case of uncoordinated sensors monitoring the same process (or correlated metrics of the same process) or in the simplest case of redundancy. The type and nature of the neighborhood can be described in two ways: logical, which happens if the nodes in the neighborhood are those measuring metrics with strong correlations [17,18], or physical, when it is present a spatial redundancy of the metrics tracked (e.g., temperature or humidity in various points of a room). Regardless of the nature of the neighborhood and the reason for the correlation, it is clear that when a sensor collects and transmits data, these updates can also be useful to its neighbors. Finally, AoI assumes underlying binary information. In reality, information coming from sensors, especially tracking smart living applications, can be multi-structured [19,20], and an interpretation is often required.

In light of the aforementioned points, in this paper, we investigate how during the acquisition of information by a WSN the correlation inside the data can improve the AoI. More precisely, we studied a scenario where every sensor can send an update with probability *p* (and thus reset its AoI); furthermore each one of this update has a probability *q* to be useful to the neighbors as well (i.e., reset their AoI). We investigate how this is impacted by the numerical values of *p*, *q*, the size of the neighborhood *N*, and the transmission scheme (i.e., concurrent or time-division multiple access). We show both theoretical and numerical results, proving the potential advantages of including AoI in the scheduling policies for WSN, especially for resource-constrained applications.

Furthermore, we study how ML algorithms can influence these scenarios. As mentioned above the AoI not consider the intrinsic value of the update. However, the data collected from multiple sensors can be multi-structured, i.e. multidimensional and heterogeneous, and ML can help us to extract meaningful information that can be handled in the updates [21,22,23,24]. Using these techniques can bring both benefits and disadvantages for smart living ecosystems. A strength it is the ability of the these algorithms to combine information from multiple sources that perform different measurements and exploit the correlation among the data. This could lead to a decrease of the number of updates, decrease the redundancy of the system and eventually limit energy consumption and the battery drain of the remote sensors. The other side of the coin is the risk of error propagation within the whole system due to mis-classification in the learning procedure [25].

To better highlight the novelties introduced by our work, in Table 1 it is possible to find a comparison of the topics covered in this paper versus the topics covered in other similar studies. It is possible to observe from the table these topics have already been covered before, but, to the best of our knowledge, this is the first work that tries to integrate them into a single work.

The rest of the paper is divided as follows. Section 2 presents the scenario we want to investigate. Section 3 presents the analysis and the results regarding the evolution of the AoI from correlated sources for two different scenarios. Section 4 analyzes how ML can interact with an AoI-based system. Finally, Section 5 drives the conclusions and suggests some interesting future work.

## 2. Scenario and Methodology

Consider a smart living environment monitored by a WSN of *N* sensors, i.e., belonging to set N={1,2,⋯,N}, that samples information and sends it to a central server S, where it is processed and analyzed. Time is discrete, i.e., $t\in Z^{+}$, and in each time slot a sensor can decide to sense new information from the environment and send an update to the central server. The sensed information may be correlated at different locations. We aim to take advantage of this correlation to decrease the number of useless transmissions but keep the average AoI as low as possible [15,16,27]. Particularly, in each time slot, we consider either of the following two possibilities: a sensor, e.g., sensor 1, senses a new sample of information and transmits the fresh sample to the central server, and this event is assumed to happen with a probability equal to *p*. Or, any other sensor acquires a new sample and sends an update. This update can be useful for sensor 1, too, and this event is assumed to happen with a probability equal to *q*. The sensor’s AoI is reset either when it transmits, or when the transmission of one of its neighbors is useful to it. In addition, we assume that all sensors are characterized by the same values for *p* and *q*. In the following, we consider two different medium access strategies, i.e., concurrent and time-division multiple access (TDMA [34]) and we study the behavior of the average AoI in time as the parameters *p*, *q* and *N* vary.

Later, we introduce the use of ML to optimize the policy of updating the AoI of each individual sensor, provided that it is used to possibly identify anomalies in the environment. We study how the misclassification probability ($p_{\mathrm{err}}$) of the ML algorithm and the other parameters of the model, i.e., *p*, *q* and *N*, influence the average AoI and the number of transmission ($N_{TX}$).

For convenience, the list of the notation used in this article is available in Table 2.

## 3. Multiple Access

### 3.1. Concurrent Multiple Access

In this scenario, the sensor nodes are allowed to transmit data in any possible time slot, without prior coordination with the other nodes. Particularly, at each time slot, the probability that a sensor transmits is *p*. We investigate the behavior of the system in this setting using a Markov chain to model the average AoI of a sensor with a variable number of neighbors *N* [16], especially in case of poorly or strongly correlated information coming from different locations, i.e., sensors. The states of the Markov chain are used to model the AoI of a sensor and the transition represents its increase or decrease. In each state two possible outcomes are possible: the sensor does not transmit and the AoI increases, so the model goes the the next state. Alternatively, the sensor transmits, or a neighbour transmits useful data, and the model returns to the initial status with value 0. Computing the steady-state probability of the Markov chain enable us to evaluate the average AoI of the system.

**Figure 1:**
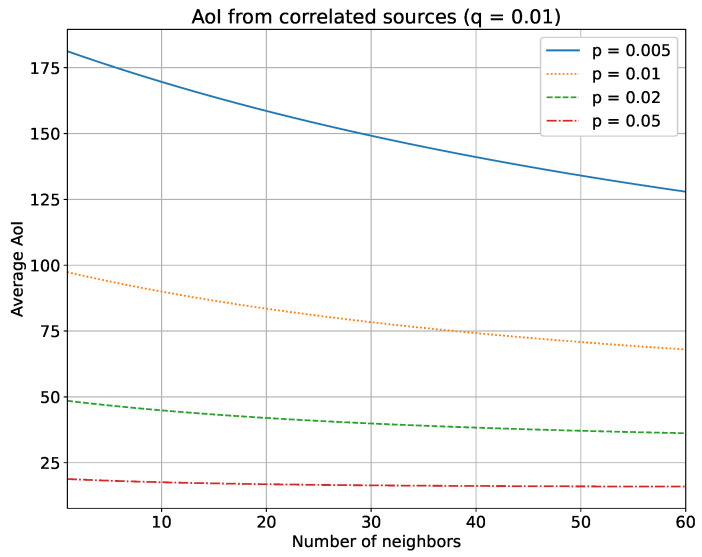
Behavior of the average AoI with a variable number of neighbors (*N*) in a loosely correlated scenario (q=0.01) with the concurrent access scheme.

**Figure 2:**
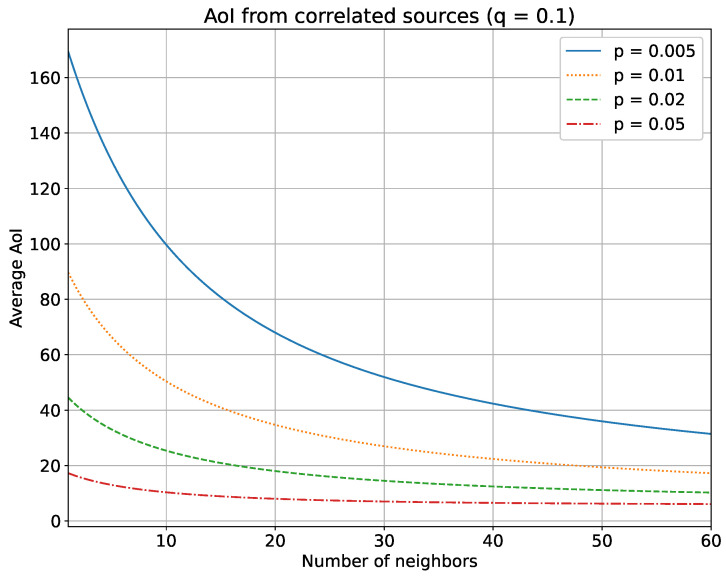
Behavior of the average AoI with a variable number of neighbors (*N*) in a strongly correlated scenario (q=0.1) with the concurrent access scheme.

We report our main findings in Figure 1 and Figure 2. They show the relationship between the average AoI and a variable number of neighbors (*N*) in a loosely correlated scenario (with q=0.01), or in a strongly correlated scenario (with q=0.1).

As one might intuitively expect, the average AoI drops as the number of neighbors increases. Noteworthy is the fact that the decrease is much more evident for low probabilities of transmission (blue continuous line). This is due to the fact that when a node updates more frequently, any contributions from its neighbors become marginal. Instead, for lower values of *p*, the gain from neighbors’ updates is larger. It is worth noting that this behavior implies that increasing the number of neighbors is beneficial up to a certain value, depending on *p*, and after which each additional neighbor no longer contributes to decreasing the system’s AoI (e.g., in Figure 2 the AoI remain practically flat for any number of neighbors N>20). Furthermore, as might be expected, the decrease is much more visible in the scenario with a strong correlation.

This can be leveraged whenever we want to reduce the energy consumption of the sensors without significantly affecting the AoI. In fact, based on this simulation, with a high enough number of neighbors, we can keep *p* as low as possible (i.e., sparse updates, low number of transmissions), while having a low AoI, too. Consequently, the battery life of the sensors can be prolonged, since decreasing the number of updates means fewer transmissions, thus lower energy consumption. At the same time, few transmissions mean low network overload and this can additionally reduce the likelihood of collisions due to wireless media and the consequent loss of data.

To note, in this scenario, we did not consider possible collisions from simultaneous transmissions. The model can be promptly extended to take into account collisions and re-transmissions, which is already investigated in the literature [6,35,36].

### 3.2. Time-Division Multiple Access

TDMA is an instance of deterministic multiple access that entirely avoids concurrent transmissions [34], which is useful in case sensors are allowed to transmit only in their assigned time slot. Each transmission cycle accounts for a certain number of time slots τ, and different scheduling strategies can be realized for ordering the transmissions of the sensors. In this work, we consider a simple round-robin scheduling where the sensors are polled by the sink (i.e., the server) in sequential order: for example, sensor 1 is allowed to transmit only in the time slots t=0,Nτ,2Nτ,⋯, while sensor 2 can transmit at t=1,1+Nτ,1+2Nτ,⋯, and so on. In general, sensor *j* can transmit in slot *k* if and only if (kmodN)=j−1. In each of its allowed transmission opportunities, a sensor transmits a new sample with a probability *p*. In addition, similarly to the scenario with concurrent access (see Section 3.1), the probability that the new acquisition of a neighbor is helpful for a sensor to reset its AoI is equal to *q*.

In the following, we study this scenario through both a theoretical formulation and numerical simulations.

Particularly, we study the problem of computing the average AoI of the time-division system via theoretical formulation, i.e., obtaining a closed-form expression for the expected value of AoI of a sensor in the network. Given the assumption that all sensor nodes share the same *p* and *q*, that is $p_{i}=p$ and $q_{i}=q$, for all i∈N (symmetry assumption), the expected value of the AoI of the system (average AoI) is equal to the average AoI of any individual sensor. We consider the initial condition $t_{0}=0$ and *N* sensors. Since each sensor transmits only in its slot with a round-robin scheduling, the expected AoI can be written as

(1) $$\begin{array}{c} \begin{array}{cc} E[\mathrm{AoI}] & =\sum_{i=0}^{\infty }i\rho \left(i\right)=\sum_{j=0}^{\infty }jN\rho \left(jN\right)+\;\sum_{\begin{array}{c} k=0\\ k\neq nN \end{array}}^{\infty }\;k\rho \left(k\right), \end{array} \end{array}$$

where *i* is the value that AoI takes at time *t* for a sensor, and ρ(i) is the probability that AoI takes that specific value. Intuitively, the first term corresponds to the contributions given by a sensor to the average AoI, i.e., accounting for its transmissions in its assigned slots, while the second term represents the contributions of the other sensors during their turn (corresponding to those *t* that are not integers multipliers of *N*).

Assuming that a certain sensor accumulates an AoI of jN in the case in which it has not transmitted in any previous time slot, and no neighbor has helped with their transmissions in any of the previous time slots (intermediate time slots between the slots assigned to the sensor), we can write the first term of (1) making explicit use of the probabilities *p* and *q*, as follows:

(2) $$\begin{array}{c} \begin{array}{c} \sum_{j=0}^{\infty }jN\rho \left(jN\right)=\sum_{j=0}^{\infty }{\left(1-p\right)}^{j}\cdot {\left(1-qp\right)}^{j(N-1)}\cdot p\cdot \left(Nj\right)=\\ =\sum_{j=0}^{\infty }r^{j}\cdot p\cdot \left(Nj\right)=pN\sum_{j=0}^{\infty }r^{j}j=\frac{pNr}{{\left(1-r\right)}^{2}}, \end{array} \end{array}$$

where $r=\left(1-p\right)\cdot {\left(1-qp\right)}^{\left(N-1\right)}$. Thus, we are able to obtain a power series that can be solved in closed form.

Similarly, we further manipulate the second term of (1) to obtain the following:

(3) $$\begin{array}{c} \begin{array}{cc} \sum_{\begin{array}{c} k=0\\ k\neq nN,n\in N^{+} \end{array}}^{\infty }k\rho \left(k\right) & =\sum_{k=0}^{\infty }\sum_{n=1}^{N-1}{\left(1-p\right)}^{k+1}\cdot {\left(1-qp\right)}^{kN+n-1}\cdot qp\cdot \left(kN+n\right)=\\ \; & =B\left(\frac{Cs}{{\left(1-s\right)}^{2}}+\frac{D}{1-s}\right), \end{array} \end{array}$$

where

(4) $$\begin{array}{c} B=\frac{qp(1-p)}{z},\;C=\frac{N(z-z^{N})}{1-z},\\ D=\frac{\left(N-1\right)z^{N+1}-Nz^{N}+z}{{\left(1-z\right)}^{2}},\\ s=\left(1-p\right)z^{N},\;and\;z=\left(1-qp\right). \end{array}$$

**Figure 3:**
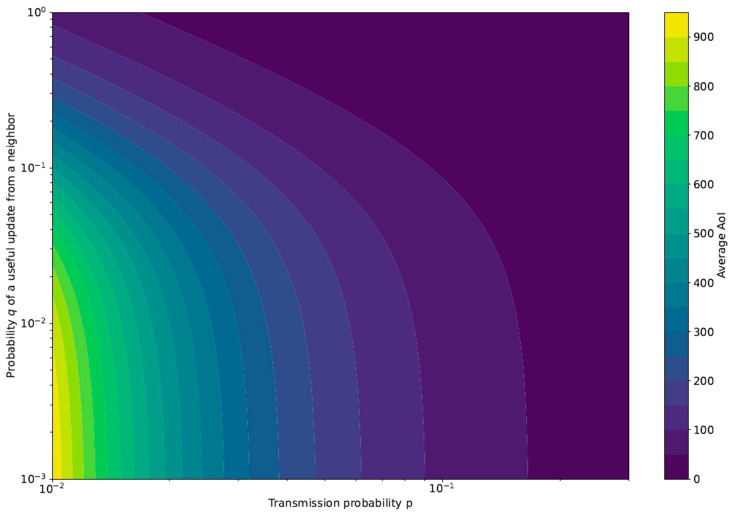
Average AoI obtained from the theoretical framework with N=10.

The second term accounts for the transmissions with index equal to *k*, with *k* not an integer multiplier of *N*, i.e., the transmissions of the neighbors. For the AoI to reach the value (kN+n), all previous transmissions by the sensor and all its neighbors have to be either missed or not useful. Figure 3 shows the AoI behavior in terms of both *p* and *q*. As expected, the average AoI is maximum for low *p* values, i.e., rare transmissions of the sensor, and poor correlation with the neighbors, i.e., low *q* values.

**Figure 4:**
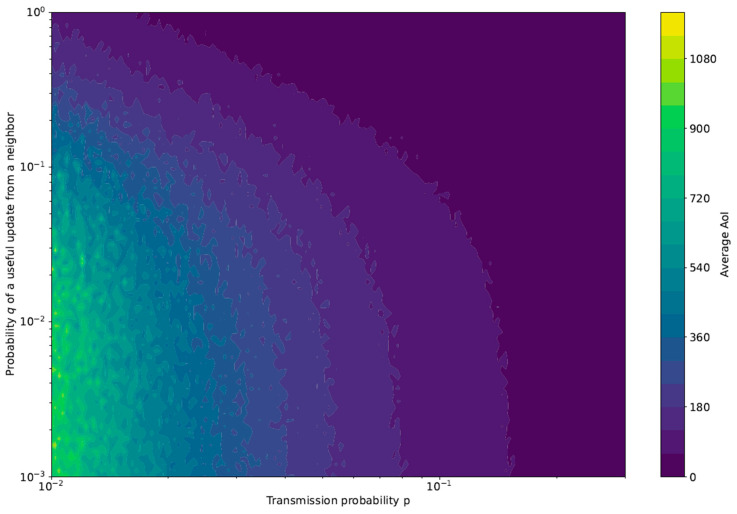
Average AoI obtained from simulations with N=10.

To further validate our theoretical framework, we ran simulations of the same scenario using Python version 3.8.15. Each simulation ran for $10^{6}$ iterations, i.e., time slots, and the AoI was computed for every sensor. The average AoI of every individual sensor was obtained. Finally, the average AoI of the system was computed by taking the mean value of the average AoI among all sensors. Figure 4 shows the AoI behavior in terms of both *p* and *q*. This figure corresponds to Figure 3, obtained through the theoretical framework. As expected, the simulations confirm the theoretical analysis, with just minor numerical discrepancies.

**Figure 5:**
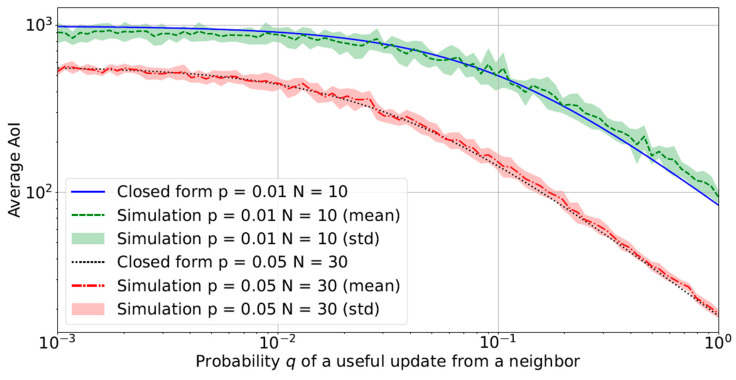
Average AoI with a variable probability of useful updates *q* from a neighbor. Simulation and theoretical results are overlapped.

**Figure 6:**
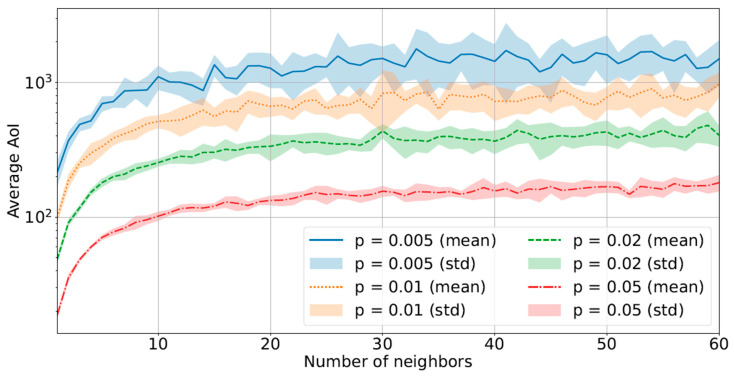
Average AoI with a variable number of neighbors in a strongly correlated scenario (q=0.1).

**Figure 7:**
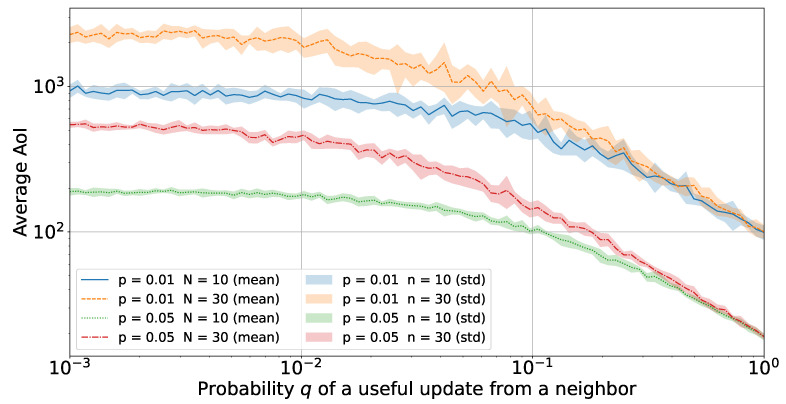
Average AoI with a variable probability *q* during the simulation of the time-division multiple access for various values of *p* and *N*.

Through simulation, we also investigate the impact of the main parameters of the model, i.e., *p*, *q*, and *N*. The results are shown in Figure 5, Figure 6 and Figure 7. For all parameter combinations we ran multiple simulations and reported the mean and standard deviation in the various figures.

Figure 5 shows the average AoI with a variable *q* for two particular combinations of *p* and *N*. First, the figure shows the full agreement between simulations and theory. Second, as we might expect, AoI is significantly decreased by increasing the probability of transmission of each sensor (*p*) and the number of neighbors (*N*). Third, the advantage of having higher *p* and *N* is more evident when the correlation between nodes decreases. For poorly correlated nodes (e.g., q≤0.01), the average AoI is high, while for strongly correlated sensors (e.g., q≥0.1), choosing the setting with higher *p* and *N* might produce a decrease of an order of magnitude in the AoI.

To better quantify the importance of *p* for AoI, in Figure 6 we show the AoI differences when choosing different values of *p*, spanning over an order of magnitude (from $5\times 10^{-3}$ to $5\times 10^{-2}$) with a variable number of neighbors. We can observe that there is a significant decrease in AoI when *p* consistently increases. In the case of strongly correlated sensors, the difference is stable no matter the number of neighbors.

It is also worth noting that the higher *N*, the higher the average AoI, in contrast with the case of concurrent access (see Figure 2). This is due to the access scheme to the medium used. The correlation between the nodes undoubtedly allows the nodes to exploit the transmissions of the neighbors to obtain fresh information samples without the need for your own acquisitions but the sensors are still forced by the time-division scheme to wait an entire cycle to transmit again. The duration of the cycle grew linearly with the number of nodes in the network and therefore, despite the benefit given by the correlation, the increase in the number of nodes is actually counterproductive. For the same reason, this finding remains true also in the case of sensor nodes within a WSN with a relatively high correlation factor (i.e., q≃0.1). The increase in the time delay between the transmissions, caused by the round-robin scheduling in the time-division scheme, cannot by compensated by the high correlation and the only viable option to decrease the AoI remain the increase of the transmission probability (*p*). To note that with p=1, with this scenario the AoI will be a function of *q* with value between *N* (q=0) and 1 (in the case q=1).

Finally, Figure 7 offers new insights into the impact of *q* on the time-division scheme. It represents the trade-off between *q* (level of correlation among nodes) and *N* (number of nodes in the network): when *q* is sufficiently large (q≥0.1), there is no gain in increasing the number of neighbors. To decrease AoI, it is more convenient to decrease *p*. This can be regarded as one of the most favorable conditions, i.e., the high correlation between a sufficiently high number of neighbors leads to the possibility of reducing the number of transmissions of every single node and does not necessarily imposes to increase the number of nodes in the network. In addition, for a poorly correlated scenario (q≤0.01), the number of neighbors *N* and the transmission probability *p* tend to dominate the behavior of AoI, thus providing a flat AoI curve. As *q* increases (0.01<q<0.1), its impact on AoI becomes larger, and for a highly correlated scenario (q≥0.1), the AoI tends to converge to 1/p, independently of *N*.

## 4. ML-Based Sensor Transmission Optimization Using AoI

In real world scenarios, the information coming from sensors can be multi-structured and data can have different importance levels for the end user [19,27]. The application of ML offers a powerful tool to integrate this aspect and extend the concept of AoI in the more general concept of ”value of information” where the semantic aspects of the data become important to decide whether to transmit them, or not.

In our considered scenario, the *N* sensors can adjust their update rates based on how fresh is the information they deliver to the destination. Furthermore, ML algorithms can be used to analyze the data and classify each update as *normal status* or *anomaly*. This adds a further processing step to the system and might lead to different possible results. For example, the update can carry no important information (*normal status*), so the AoI for that process can be updated less frequently, to save power and keep bandwidth free for other transmissions. Alternatively, an alarm needs to be raised (when an *anomaly* is detected), and AoI must be kept very low, i.e., the update rate increase, at the cost of a temporary higher energy consumption [37]. Finally, the update can be inconclusive. This happens when the content of the update is not clear, so old data keeps being used, with an AoI value that is increased by 1.

However, such an ML-based approach is sensitive to classification errors [38]. For example, there can be an apparently valuable update (some *anomaly* status that requires immediate action), which is actually a false positive, i.e., it is a normal status that the algorithm that the ML-algorithm misclassified. This error has little impact on the system as the only outcome is an extra transmission from a sensor that monitor a process where there are no anomalies at that specific moment. Still, energy is wasted, which may lead to inefficiency at the ecosystem level. On the other hand, if no valuable update (*normal status*) is reported when an anomaly is actually occurring (false negative), the problem is more relevant [25]. This condition should be carefully monitored with frequent updates, but the sensor has no reason to maintain its AoI low, and thus, continues its routine (i.e., *normal*) operation possibly leading to a damage for the entire system.

One possible solution to increase the robustness of this ML-based approach is to use ML to aggregate different measures taken over time, instead of simply classifying each update. Aggregating different measurements through some principles of participatory federated learning [28] can lead to a beneficial holistic view of the entire system. In particular, system-wide anomalies can be identified and in the end a more accurate classification is provided, also based on historical records [24].

**Figure 8:**
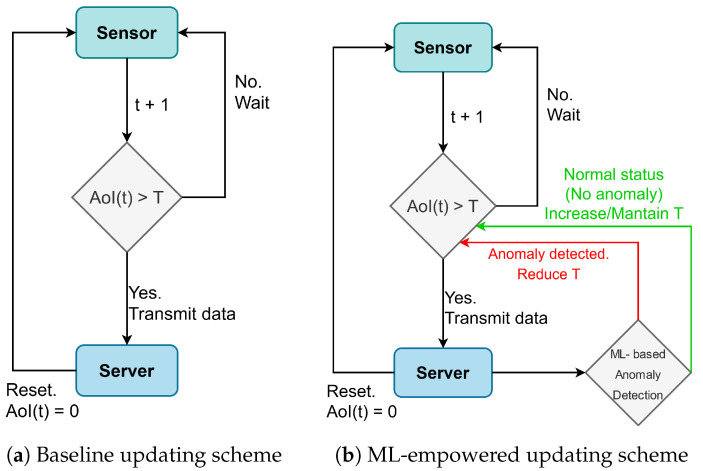
The role of ML in the sensor’s AoI optimization. A baseline scheme without ML (**a**) is compared with an ecosystem with ML in the loop (**b**), with a dynamic adjustment of the AoI policy (i.e., updating a threshold *T*).

In the following, we explore the adjustment of AoI operating policies according to the actual content of the updates [23] using ML. We compare a baseline scheme, where an update is sent whenever AoI is greater than a predefined threshold *T*, with a scheme where ML is used to classify the updates into *anomalies* or *normal* data, so that the value of *T* is updated accordingly, e.g., to give higher priority to signaling anomalies. A logical scheme of this comparison is shown in Figure 8.

### Results

As just mentioned, to assess the impact of ML, we simulated the behavior of a single sensor tracking the average AoI and the total number of its transmissions. We simulated two scenarios, one without ML (henceforth referred to as the *baseline* case) and one with a ML for classifying the received updated. We did not actually consider a specific ML scheme, but we accounted for the misclassification events and the possibility of aggregating and leveraging information from neighbor nodes.

The simulation considers a discrete time axis with $10^{4}$ time slots. The status of a single sensor and its AoI are tracked at each time step, with 4 possible outcomes: (i) the sensor sends an update with probability *p*. Therefore, AoI is reset to 0 and the number of transmissions is increased by 1; (ii) at least one of the *N* neighbors sends a useful update with probability *q*. Therefore the AoI of the sensor is reset but the number of transmissions is not increased; (iii) the AoI exceeds the predefined value *T* (set at the beginning of the simulation to some quantity $T_{0}$) and the sensor is forced to send an update, so that once again the AoI is reset to 0 and the number of transmissions is increased by 1; (iv) none of the previous cases, so no update is performed. In this case the AoI is increased by 1, but the number of transmissions from that sensor is kept the same.

Each update is supposed to be classified through a ML algorithm into a binary outcome (*normal status* or *anomaly*), with a symmetric probability of misclassification being equal to $p_{\mathrm{err}}$. According to our previous description (see Figure 8), we modified the AoI threshold according to how the ML procedure classifies the update. In particular, the initial threshold is set to $T:=T_{0}$; then, whenever an anomaly is detected, the threshold is set to max(1,T/2) to force the system to sending more frequent updates (ideally, every slot if the anomaly persists). Otherwise, the threshold is increased by 1, so $T=\min(T+1,T_{0})$.

**Figure 9:**
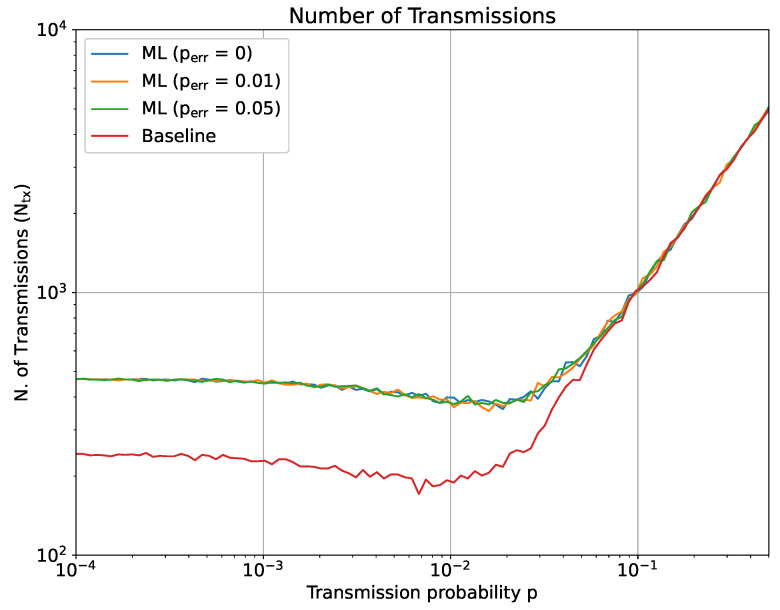
ML-based optimization of the sensor’s AoI: total number of transmissions after $10^{4}$ time slots.

**Figure 10:**
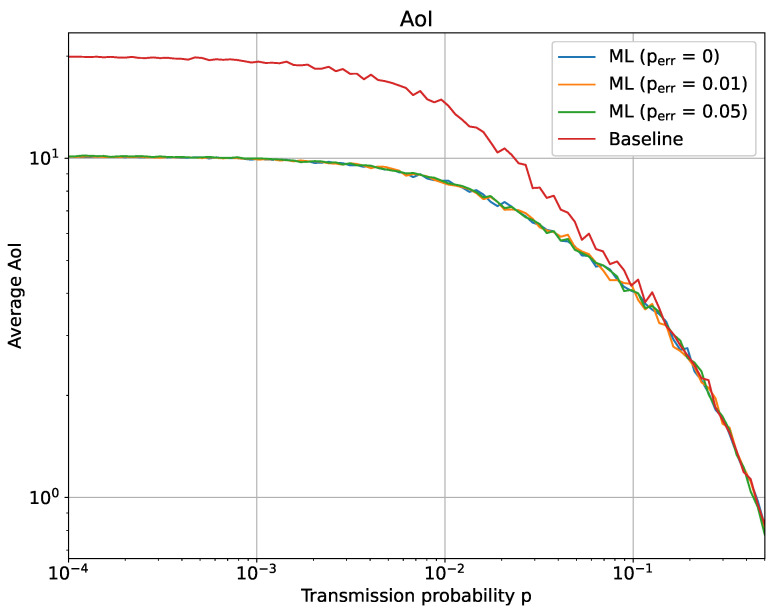
ML-based optimization of the sensor’s AoI: average AoI after $10^{4}$ time slots.

We simulated this scenario for different values of *p*, $p_{\mathrm{err}}$, *N*, *q* and $T_{0}$. Figure 9 and Figure 10 show two representative results in the case N=30, q=0.15 and $T_{0}=30$. Incidentally, we notice that the results do not significantly differ for different choices of those parameters. In particular, Figure 9 shows the number of transmissions ($N_{tx}$) with a variable transmission probability *p*, while Figure 10 reports the AoI behavior when *p* ranges between $10^{-4}$ to 1.

As can be observed from both figures, the effect of ML is more evident for lower values of *p*. For lower transmission probabilities, the baseline scenario obtains an average AoI and a number of transmissions that are only influenced by $T_{0}$, since the only way that the AoI is reset to 0 is when the sensor is forced to update after hitting $T_{0}$. In this same situation, the impact of an ML-empowered tracking is to decrease the number of transmissions, since it allows to exploit the redundancy present from the network structure, but also consequently implying a slight increase in the average AoI. This effect vanishes, as it might be expected, with the increase of the transmission probability. No relevant differences can be noted for the $p_{\mathrm{err}}$ tested, thereby implying that a limited error rate can be recovered thanks to subsequent correct updates. It is interesting to note that the number of transmissions decreases for $p\leq 10^{-2}$, while rapidly increasing after this particular values. Further tests (not reported due to space constraints) showed that a similar behavior occurs for different values of *q* and *N* too; however, it becomes more evident for larger *q* and *N*, while it almost vanishes when *q* and *N* are sufficiently small. Overall, we might explain this behavior with the fact that the correlation between the sensor in this range of *p* ($p\leq 10^{-2}$) dominated the system’s AoI and total number of transmission. With the increase of *p*, the correlation between the sensors becomes a weaker contribution to the AoI, compared to the simple increase of transmissions for each sensor.

For a scenario with sparse update, it is possible to conclude that ML-empowered algorithms can be exploited to reduce the number of transmission ($n_{tx}$) and consequently energy consumption of the sensors and network congestion. The downside to applying these techniques lies in the increase in AoI and makes the system more exposed to possible failures if critical updates are misclassified. Yet, the possibility of collecting and combining data from multiple sources and/or time instances may lead to richer description of the system status and avoid this problem. Future tests in more extended setups, and possibly in real world scenarios, will be needed to find the adequate trade-off between reducing the number of transmissions and the choice of the specific ML scheme to adopt.

## 5. Conclusions and Future Work

In resource-constrained environments, the availability of fresh information is an important challenge that can be addressed through AoI. In this paper, we showed how exploiting the correlation between multiple sources of information in the computation of AoI, beyond its standard definition, can be beneficial to lowering the AoI and keeping the system up-to-date. At the same time, we showed how the transmission protocol can strongly influence the AoI, which can even increase despite the exploitation of correlation among multiple sources. Furthermore, we showed the importance of applying ML-empowered classifications of the state of the ecosystem, thus using the semantic value of the complex data collected by the sensors to adjust the AoI. In the future, the proposed approaches aiming at enriching the representative value of AoI could be tested in different real-world scenarios, in order to test it and adapt it to the specifics of different applications.

## Figures and Tables

**Table 1:** Coverage of the topics of our paper from various studies.

	AoI	Energy	Correlation	Machine Learning	Transmission Policies
Bacinoglu et al. [10]	X	X			X
Wu et al. [12]	X	X		X	X
Kalor and Popovski [15]	X		X		X
Safdar and Do-Hyun. [3]		X		X	
Zhou and Saad [14]	X		X		X
Samir et al. [22]	X	X		X	
Jin et al. [26]	X	X	X		
Fountoulaki et al. [8]	X	X			
Badia [9]	X		X		X
Crosara and Badia [11]	X	X			X
Zancanaro et al. [16]	X		X		
Elgabli et al. [21]	X			X	
Crosara et al. [27]	X		X		
Bellavista et al. [28]			X	X	
Ceran et al. [29]	X			X	X
Wang et al. [30]	X	X		X	
Fang et al [31]	X	X		X	
Tong et al. [32]	X		X		
Shiraishi et al. [19]	X	X		X	X
Zancanaro et al. [33]	X	X	X	X	
**Our work**	X	X	X	X	X

**Table 2:** List of symbols used in the article (in order of appearance in the following).

Notation	Definition
**Multiple Access (Section 3)**	
*t*	time slot index
*N*	no. sensor nodes
*p*	transmission probability of every sensor
*q*	probability of useful transmission from a neighbor node
τ	duration of a time slot
ρ(i)	probability that the AoI has value *i*
**ML-based AoI optimization (Section 4)**	
$N_{TX}$	no. transmissions
$T_{0}$	initial AoI threshold for the ML simulation
*T*	AoI threshold during the ML simulation
$p_{err}$	probability of mis-classification for the ML algorithm
